# Metabolic Responses in Leaves of 15 Italian Olive Cultivars in Correspondence to Variable Climatic Elements

**DOI:** 10.3390/plants12101953

**Published:** 2023-05-11

**Authors:** Ilaria Colzi, Elettra Marone, Simone Luti, Luigia Pazzagli, Stefano Mancuso, Cosimo Taiti

**Affiliations:** 1 Department of Biology, University of Florence, Via Micheli 1, 50121 Firenze, Italy; 2 Department of Biosciences and Technologies for Agriculture, Food and Environment, University of Teramo, Via R. Balzarini 1, 64100 Teramo, Italy; emarone@unite.it; 3 Department of Biomedical Experimental and Clinical Sciences, University of Florence, Viale Morgagni 50, 50134 Firenze, Italy; simone.luti@unifi.it (S.L.); luigia.pazzagli@unifi.it (L.P.); 4 Department of Agri-Food and Environmental Science, University of Florence, Viale delle Idee, 50019 Sesto Fiorentino, Italy

**Keywords:** olive leaves, climactic variations, oleuropein, volatile organic compounds

## Abstract

This study aims to evaluate the metabolic changes that occurred in olive leaves as responses over time to variations in climatic elements. Rainfall, temperature, and solar radiation data were collected over 4 months (August–November) to assess the impact of different climatic trends on the metabolism of the leaves of 15 Italian olive cultivars, cultivated at the experimental farm of the University of Florence. The net photosynthetic rate (A_N_) and stomatal conductance (g_s_), measured as main indicators of primary metabolism, were mainly influenced by the “cultivar” effect compared to the “climate” effect. The lowest A_N_ value was showed by “Bianchera”, while “Ascolana” recorded the highest (8.6 and 13.6 µmol CO_2_ m^−2^s^−1^, respectively). On the other hand, the secondary metabolism indicators, volatile organic compound (VOC) and oleuropein (OL) content, were much more influenced by climate trends, especially rainfall. A phase of high rainfall caused a significant increase in the VOCs emission from leaves, even with different behaviors among the genotypes. The highest differences were observed between “Maiatica di Ferrandina”, with the highest average values (~85,000 npcs), and “Frantoio”, which showed the lowest (~22,700 npcs). The OL content underwent considerable fluctuations in relation to the rainfall but also appeared to be controlled by the genotype. “Coratina” always showed the highest OL concentration (reaching the maximum ~98 mg g^−1^), indicating the great potential of this cultivar for the industrial recovery of OL.

## 1. Introduction

The vegetal tissues and organs of perennial plants, such as the leaves of the evergreen cultivated olive (*Olea europaea*, subsp. *europaea*, var. europaea Green), are adapted to variations in climatic elements (e.g., temperature, rainfall, solar radiation). Much evidence indicates that environmental variables have a great influence on plant primary metabolism and, subsequently, on a plant’s secondary metabolites, primarily due to changes in the transcription factors responsible for their synthesis [1]. Therefore, climatic conditions represent potential factors in changing the pathways of several secondary metabolites from different families, such as volatile organic compounds (VOCs) and the green leaves volatiles (GLVs), largely spread in higher plants. However, except for that which has been reported by some authors [2,3,4], a lack of information exists on the VOCs emission by olive leaves and, in particular, on the comparison of the emission among different olive cultivars. Likewise, the studies on biophenols (BPs) in olive tree leaves are focused on the different factors (biotic and abiotic) influencing the amount, the concentration, and the pharmacological proprieties of these antioxidant compounds, although climatic conditions are still scarcely investigated [5,6]. Oleuropein (OL) is a biophenol compound distinctive of all species of genus *Olea* [7] and is considered the major BP extractable from the olive tree leaves [8]. Regarding OL, a specific review has been presented on the pharmacological effects and potentiality of this compound [9], and more recent studies have better highlighted the role of this molecule on human health [10]. Beyond the health effects of OL, in the past, olive leaves were used as a maker of taste and flavor when overripe fruits needed to be reinforced to produce an edible and stable olive oil. Moreover, the use of OL was suggested for improving the refined pomace olive oils [11]. The OL content in olive leaves is directly affected by some factors, such as agronomic conditions [12], genotype [5,13], fruits load [14], and leaf age [15,16,17]. In particular, Yazici et al. (2012) [12] showed that the OL content was influenced by water availability (irrigation), while Martinez-Navarro et al. (2022) [18] investigated the changes in OL content in Arbequina cv determined by crop management, plantation framework, and climate elements (humidity and temperature). However, it is not yet clear what effect the climatic events (temperature, solar radiation, and precipitation) may have on the OL content in different olive cultivars. Moreover, due to the growing scientific and economic interests related to the use of olive leaves and their extracts [19,20], the dynamic of the OL content in leaves requires more accurate knowledge in relation to leaf age, genotypes, and climatic elements. 

A key goal of this study was, therefore, to investigate the interplay between the environment and some aspects of olive plant metabolism, both primary and secondary, and, in particular, how climatic factors can affect some metabolite concentrations (e.g., VOCs and OL). It is known that the genotypeXenvironment interaction is mainly due to the variation of meteorological elements, and that it is characterized by the differential response of each genotype [1]. The characterizing of primary metabolic mechanisms, such as plant gas exchange processes (e.g., photosynthesis and water-vapor transfer), is fundamental to understanding the general plant response to environmental variations [21], since these processes respond very rapidly to external factors and their measurement provides immediate indications on the performance of different genotypes [22,23]. Likewise, the synthesis of plant secondary metabolites can also be influenced, positively or negatively, by the environment. Therefore, more knowledge is required to identify how, and which, climatic factors could mainly affect the amount of olive leaves’ BPs. Such information is particularly relevant considering that extreme weather events (both droughts and floods) driven by the intensification of the global water cycle are increasing their frequency by threatening the agrifood production and security [24]. Moreover, the identification of genotypes more tolerant to such climatic variations may help in minimizing the downside risk associated with future climates [24].

This study was carried out on rain-fed plants of 15 different cultivars with the following objectives: (1) to compare the possible sensitivity of different olive genotypes to the variability of the main climatic elements (temperature, solar radiation, rainfall) in short and defined time spans by monitoring two main indicators of the primary metabolism, the net photosynthetic rate (A_N_) and the stomatal conductance (g_s_) recorded over time; (2) to determine the response of selected genotypes to the variation of the main climatic elements (temperatures, solar radiation, rainfall), by examining the spectra fingerprint of VOCs emission from wounded leaves; and (3) to individuate, among the different examined genotypes, those presenting the higher amounts of OL in 1–2 year-old leaves, and the variation that can be determined by the main climatic elements which could occur during a specific growth season, here autumn. 

## 2. Results

### 2.1. Leaf Gas Exchange Measurements

**Figure 1:**
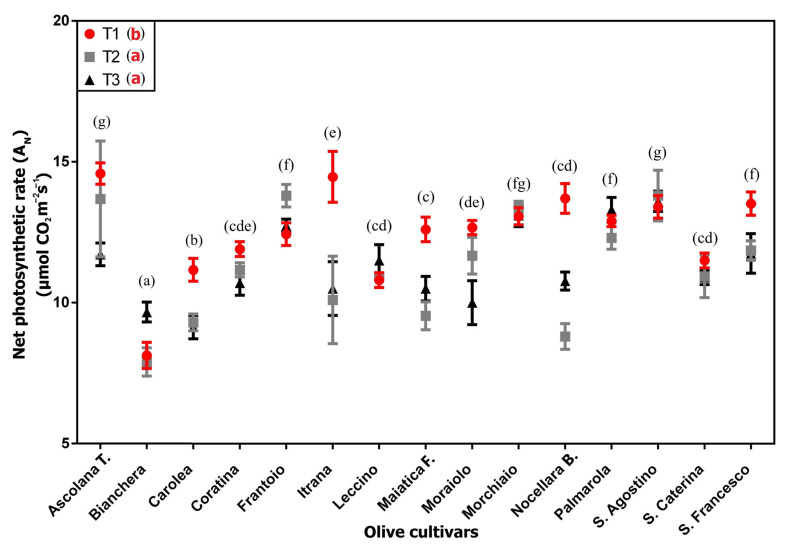
Net photosynthetic rate (A_N_, µmol CO_2_ m^−2^s^−1^) measured on 15 olive cultivars at the 3 sampling times (T1, T2, T3) (average ± s.d.). Different lowercase letters indicate the significant differences: black letters for difference among the cultivars, considering the average value of the three sampling times for each cultivar; red letters for difference among the sampling times (T1, T2, T3), considering the average value of all the cultivars at the same time.

**Figure 2:**
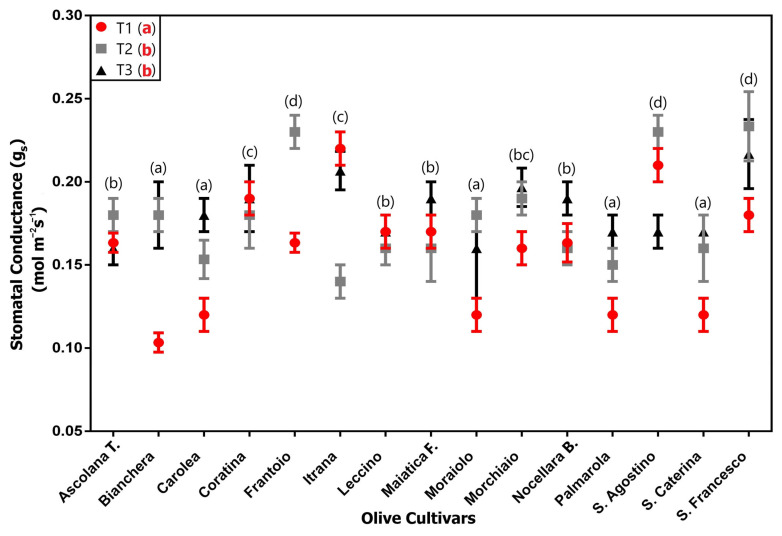
Stomatal conductance (mol m^−2^s^−1^) measured on 15 olive cultivars at the 3 sampling times (T1, T2, T3) (average ± s.d.). Different lowercase letters indicate the significant differences: black letters for difference among the cultivars, considering the average value of the three sampling times for each cultivar; red letters for difference among the sampling times (T1, T2, T3), considering the average value of all the cultivars at the same time.

Both for A_N_ and g_s_, there was a relevant variability among the different cultivars (Figure 1 and Figure 2, respectively). For both parameters the “cultivar” effect resulted in the most important contributor to the overall sum of squares, contributing 61.48% and 40.29% for A_N_ and g_s_, respectively, whereas the “time” effect resulted in 10.55% in the case of A_N_, and 12.16% for g_s_ (Table 1). A strong effect of interaction between the two factors can also be highlighted (22.8% for A_N_ and 36.9% for g_s_, respectively).

The sampling time effect indicates two opposite trends; in the case of A_N_ there is a statistically significant decrease from T1 to T2 and T3, the latter (T2 and T3) being statistically equal (Figure 1 and Table 1), while for g_s_ there is an opposite trend (Figure 2 and Table 1), from the lowest value recorded at T1 (0.16 µmol CO_2_ m^−2^s^−1^) to the highest values at T2 and T3 (0.18 µmol CO_2_ m^−2^s^−1^).

Comparing the different cultivars, the lowest average A_N_ value was showed by “Bianchera” (8.6 µmol CO_2_ m^−2^s^−1^), while the cv “Ascolana t.” and “S. Agostino” recorded the highest value (13.6 µmol CO_2_ m^−2^s^−1^), with cv “Ascolana t.” also showing the highest ever value with a peak of 15.3 at T1. The g_s_ values were much more uniform; the cv “Frantoio”, “S. Agostino” and “S. Francesco” showed the highest average values.

### 2.2. VOCs Analysis

**Figure 3:**
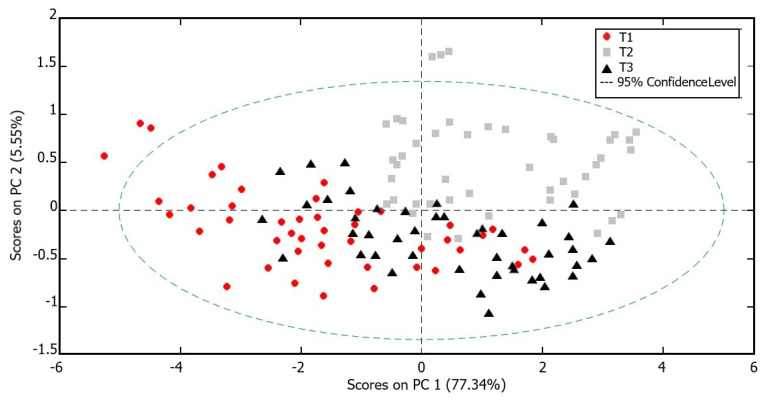
PCA ordination of the 15 olive cultivar leaf samples over time based on the VOCs full spectra.

A PCA (Figure 3) applied to the entire set of VOC data as an unsupervised ordination method allowed us to obtain a first general overview of their latent structural relationships. The total percentage variance captured by PCA based on the three components resulted of 87.79% (PC1 = 77.34%, PC2 = 5.55%, PC3 = 4.91%, respectively). In the bidimensional space of the first two components, the scores plot showed an evolution over time, even if partially overlapped. The points tend to move in the space highlighting the main influence of the factor “time” compared to the factor “cultivar”, showing three main clouds in which the cultivars are distributed. This trend is confirmed by the two-way ANOVA results (Table 2). In fact, the “time” effect resulted in the most important contributor to the overall sum of squares, contributing 33.38%, followed by the “cultivar” effect (14.95%). A strong effect of interaction between the two factors (44.59%) can also be highlighted. 

**Figure 4:**
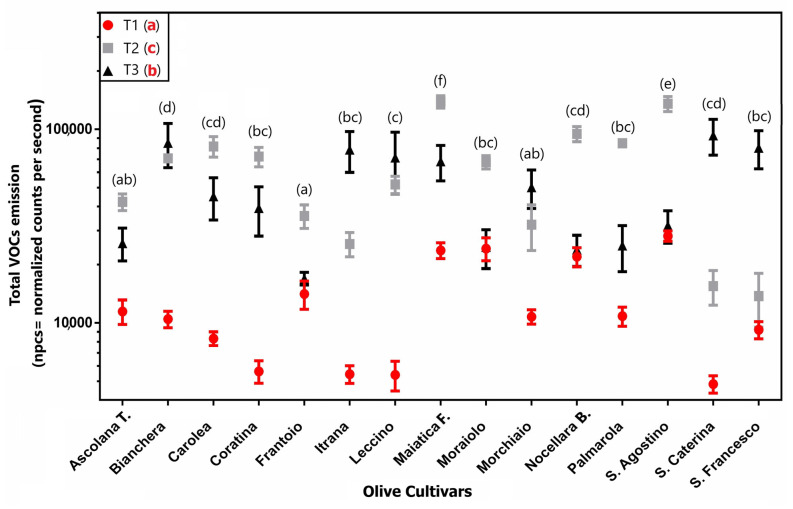
VOCs amount (ncps) obtained from 15 olive cultivars at the 3 sampling times (T1, T2, T3) (average ± s.d.). Different lowercase letters indicate the significant differences: black letters for difference among the cultivars, considering the average value of the three sampling times for each cultivar; red letters for difference among the sampling times (T1, T2, T3), considering the average value of all the cultivars at the same time.

Figure 4 reported the total amount of VOCs emitted (average ± s.d.) from the leaves of the 15 examined cultivars for each of the sampling times. It can be noted that there are important quantitative differences in the averages of the three sampling times, where T1 showed the lower volatiles emission. On the contrary, T2 showed the highest volatiles emission with huge differences compared to T1 values. For example, in “Santa Caterina” cv, the total leaf emission changed from T1 to T3 with an increase of over 80% (Figure 4). Even among the cultivars, a statistical difference among the total emission average values should be noted (calculated on the average of VOCs emitted from leaves at each sampling times). The highest differences were observed between “Maiatica di Ferrandina”, with the highest average values (~85,000 npcs), and “Frantoio”, which showed the lowest (~22,700 npcs) (Figure 4). 

**Figure 5:**
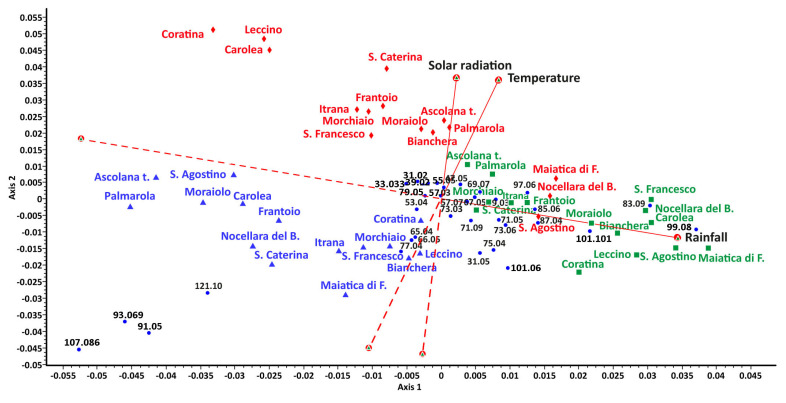
Triplot from CCoA of the VOCs data from PTR-ToF-MS. Olive cultivar leaf samples weighted average (WA) scores (red labels = T1, green labels = T2, blue labels = T3), protonated *m*/*z* (black labels), and environmental variables (temperatures, rainfalls, and solar radiation, represented by (red) arrows and black labels).

From the CCoA triplot (Figure 5), it is evident that the main role in the volatome differences was due to rainfall, which distributes the T2 samples along the red arrow with increasing correlation to it. The data of almost all the T3 samples resulted in being inversely correlated to rainfalls and were also affected in their distribution by the averages of temperatures and of solar radiation. This distribution is due to the influence that the single climatic element trends have on the volatile emission of specific compounds. For example, the masses detected at *m*/*z* = 99.090 (C_6_H_11_O^+^), *m*/*z* = 83.086 (C_6_H_11_^+^), and *m*/*z* = 101.101 (C_5_H_9_O_2_^+^) resulted in being positively correlated with the average amount of rainfall before the first sampling time (T1), whereas the masses *m*/*z* = 121.101 (C_7_H_13_^+^), *m*/*z* = 93.069 (C_7_H_9_^+^), and *m*/*z* = 91.050 (C_7_H_7_^+^), seem to characterize the volatome of the leaves taken after a period of reduced rainfall (samples belonging to T3).

### 2.3. Oleuropein Content

**Figure 6:**
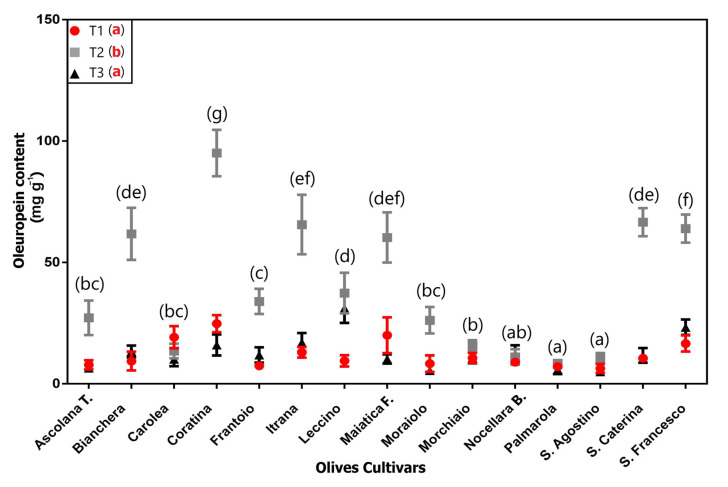
Oleuropein content (mg g^−1^) determined on 15 olive cultivars at the 3 sampling times (T1, T2, T3) (average ± s.d.). Different lowercase letters indicate the significant differences: black letters for difference among the cultivars, considering the average value of the three sampling times for each cultivar; red letters for difference among the sampling times (T1, T2, T3), considering the average value of all the cultivars at the same time.

The results concerning OL content in olive leaves of the different studied cultivars are reported in Figure 6. The OL concentration trend was similar for almost all the analyzed cultivars, highlighting highest mean values in leaves collected at T2 compared to the other two sampling times. Indeed, for most cultivars (e.g., “Bianchera”, “Frantoio”, “Maiatica di Ferrandina.”, and “S. Francesco”), the OL concentration increased in October (T2) and decreased in November (T3) before returning to September (T1) values. The only exception was represented by “Carolea”, which showed the maximum OL concentration in September (T1) and decreased in the following two months (T2 and T3). 

The two-way ANOVA (Table 3) showed that the effect of “time” factor was the most important contributor to the overall sum of squares (38.62%,) followed by the “cultivar” factor (30.16%). A strong effect of interaction between these two factors (44.59%) can also be highlighted.

For each sampling time, the OL concentration of “Coratina” was always the highest among cultivars, while the “Palmarola” showed the lowest values. In particular, the highest OL concentration was observed in “Coratina” at T2 (~98 mg g^−1^) and this coincides with the start of harvest season in Tuscany (Figure 6). The other cultivars with high values were “S. Caterina” (~66 mg g^−1^), “Itrana” (~65 mg g^−1^), “S. Francesco” (~64 mg g^−1^), “Bianchera” (~63 mg g^−1^), and “Maiatica” (~60 mg g^−1^), always at T2.

## 3. Discussion

In recent years, farming practices such as sowing date and cultivar choices have been gradually adjusted to optimize the cropping systems to weather conditions and incremental climatic change [25,26,27]. Adaptation in management, for example by selecting specific crop cultivars that are more tolerant to local conditions, can be a strategy to increase yields [28]. Several genotype (G) × environment (E) studies have been performed especially on crop species in the attempt to design models able to predict the suitable management interventions to climate change adaptation [24,28,29,30,31]. Recent studies have shown that olive trees can also be strongly affected by weather factors [32,33,34], particularly in the long term, under the Mediterranean-type climates [35]. However, not many studies have yet investigated the short-term effects of variation in the main climatic elements (temperatures, solar radiation, rainfall) on different aspects of both primary and secondary metabolisms in olive trees. 

In this study, rain-fed plants of 15 different cultivars grown in the same experimental farm were compared to evaluate the genotypeXenvironment interaction, especially in the VOCs emission and OL concentration in leaves. The possibility of analyzing cultivars grown in the same climatic and edaphic conditions is invaluable in order to better highlight possible different responses among the genotypes.

The results of the plant gas exchange measurements (A_N_ and g_s_) showed a relevant variability among the different cultivars. Indeed, despite the fact that an effect of the sampling time was observed, the main contribution to the overall variation was attributed to the “cultivar” effect. For example, “Ascolana t.” and “S. Agostino” cultivars showed the highest average values of A_N_, and “S. Agostino” also reported the highest values of g_s_. On the other hand, “Bianchera” was the cultivar with the lowest photosynthetic performance. Therefore, the set of data pointed out the fundamental role of the genetic matrix in influencing both the measured parameters of the primary metabolism, indicating a different adaptation of the genotypes to the climatic conditions. These results are comparable with earlier findings on different olive cultivars which reported considerable intra-specific variation in photosynthetic performance in response to growth conditions, such as water availability and irradiance level [36,37]. Some authors found an evident genotypic variation in drought tolerance among olive cultivars also based on a considerable variation in photosynthetic performance [38,39,40], therefore identifying the most promising ones for cultivation in arid areas [41].

If the main indicators of primary metabolism underwent a predominant effect of genotype, a different trend was instead observed for VOC emission, which seemed to be mainly affected by the sampling time. The PCA performed on the entire set of VOC data highlighted the main influence of the factor “time” compared to the factor “cultivar”. This trend was confirmed by the two-way ANOVA results (Table 2) that also pointed out a strong effect of interaction between the two factors (44.59%), highlighting the complex relationships with the environment that regulate the emission of these secondary metabolites. The three sampling times were characterized by important quantitative differences in the averages of the VOC emission, with T1 showing the lower levels and the T2 the highest ones, with huge differences compared to T1 values. The presence of such different volatiles emissions in the results was related to different climatic factors, as highlighted by the CCoA triplot (Figure 5). In particular, the main role of rainfall in the volatome differences was evident for the T2 samples, whereas the T3 samples resulted in being inversely correlated to rainfall and affected in their distribution by temperatures and solar radiation. The effect of weather conditions on VOCs emitted by olive tree leaves has never been reported, although some trends have been previously identified for olive oils. The concentration of VOCs in olive oil is indeed mainly dependent on the cultivar and the ripening stage [42,43], but some studies indicate that soil water availability is also important [42,44,45]. These studies reported that the tree water status has a great impact and major volatile compounds concentrations were higher in oils produced under irrigated conditions [44,45]. Benelli et al. (2015) [46] reported that the light environment is also a major factor inducing changes in VOCs emitted by olive oil, together with water status. Moreover, a similar effect on volatile profiles in response to weather conditions has also been observed in other plant species. For example, the fluctuation in the accumulation of volatile compounds observed during the berry development of *Vitis vinifera* was closely correlated with variations in short-term weather, especially rainfall [47].

In this work, the different distribution of VOC emission in the three sampling times was probably due to the influence that the single climatic element trends had on the volatile emission of specific compounds. The masses detected at *m*/*z* = 99.090 (C_6_H_11_O^+^), *m*/*z* = 83.086 (C_6_H_11_^+^), and *m*/*z* = 101.101 (C_5_H_9_O_2_^+^) resulted in being positively correlated with the average amount of rainfall before the first sampling time. The VOCs reported above belonged to C5 and C6 compounds developed according to distinctive biosynthetic pathways such as the “LipOXygenase (LOX) cascade” that are linked to their green fruity odors [48]. On the contrary, the masses *m*/*z* = 121.101 (C_7_H_13_^+^), *m*/*z* = 93.069 (C_7_H_9_^+^), and *m*/*z* = 91.050 (C_7_H_7_^+^), seem to characterize the volatome of the leaves taken after a period of reduced rainfall (samples belonging to T3), with a slow reduction in the average temperatures and solar radiation influence. Following the fragmentation patterns proposed by Maleknia et al. (2007) [49] for the PTR instrument, it seems that these compounds detected at *m*/*z* 91, 93, and 121, are fragments of the biggest molecules belonging to the chemical class of terpenes. According to Grubešić et al. (2021) [3] it seems that the terpene content was directly related to the phenological development of the leaves. 

Despite the strong influence of climatic factors on VOC emission, a “genotype” effect was also noted, highlighting significant differences in the volatome among cultivars. In particular, “Maiatica di Ferrandina” cv showed the highest average VOC values, whereas “Frantoio” showed the lowest ones. Moreover, “Maiatica di Ferrandina” showed the most intense response to the rainfall phenomenon observed in T2 samples, together with “S. Agostino”.

The results concerning OL content in olive leaves of the different studied cultivars showed a great variability in the average concentrations. Previous authors have already found that the genetic factor significantly impacted the content of oleuropein in the leaves of the major Italian and Greek olive cultivars [13,15], suggesting that specific genotypes may be selected as the best raw material in the industrial production of oleuropein. Interestingly, in our field study, “Coratina” was always the cultivar with the highest OL concentration, regardless of the sampling time, indicating its great potential for the industrial collection of this important secondary metabolite. On the other hand, “Palmarola” cv always showed the lowest values. 

Despite the high variability among cultivars, remarkable fluctuations in OL concentration were also found among the different sampling times, in agreement with Wang et al. (2019) [6], who observed seasonal changes in the chemical composition of olive leaves, including OL content. In fact, our results showed that the effect of “time” factor was the most important contributor (Table 3) to the overall sum of squares (38.62%,) followed by the “cultivar” factor (30.16%). A strong effect of interaction between these two factors (44.59%) can also be highlighted. In particular, the highest OL concentration was observed in “Coratina” at T2 (~98 mg g^−1^) and coincides with the start of harvest season in Tuscany. The other cultivars with high values were “S. Caterina” (~66 mg g^−1^), “Itrana” (~65 mg g^−1^), “S. Francesco” (~64 mg g^−1^), “Bianchera” (~63 mg g^−1^), and “Maiatica” (~60 mg g^−1^), always at T2. The OL concentration trend was similar for almost all the analyzed cultivars, highlighting highest mean values in leaves collected at T2 compared to the other two sampling times. Indeed, for most cultivars (e.g., “Bianchera”, “Frantoio”, “Maiatica di Ferrandina.”, “S. Francesco”), the OL concentration increased in October and decreased in November before returning to September values. The only exception was represented by “Carolea”, which showed the maximum OL concentration in September and decreased in the following two months. 

In accordance with what was previously reported by Kabbash et al. (2021) [17], the overall data indicate that there are cultivars (e.g., “Coratina”, “S. Caterina”) in which the variations in OL concentration was time-dependent and others (e.g., “Morchiaio”, “S. Agostino”, “Nocellara del Belice”) in which the OL concentration was roughly constant. Two factors of variability can be noted. The first factor influencing the OL concentration in the leaves was the collection time; for example, considering “S. Agostino” cv, the OL concentration increased in the T2 survey up to 10.8 mg g^−1^, whereas in “Coratina” cv, whose leaves are rich in OL [17], it increased up to 98.5 mg g^−1^. In the T3 survey, the OL values obtained tended towards the level of T1, highlighting a possible significant time variation of the OL concentration within homologous leaves, variations influenced by climatic factors. The second factor influencing the OL concentration variability was the great differences in the OL concentration in the dry matter of homologous leaves coming from different cultivars in the same period. In fact, for T1, there is a minimum in the “S. Agostino” (6.2 mg g−^1^), and a maximum in “Coratina” (24.3 mg g^−1^).

## 4. Materials and Methods

### 4.1. Plantation and Plant Material

Leaf samples of 15 Italian cultivars from an olive germplasm collection, grown in the Montepaldi experimental farm (43.66848433292147, 11.150554775648724, 210 m a.s.l.) of the University of Florence, were collected from 21 year-old bush-trained own rooted plants. The rain-fed orchard is planted on a soft hilly slope of sedimentarious gipsy-arenaceous soil, in a Mediterranean cold climatic zone with a rainfall season between autumn and winter. 

The vegetal material is constituted by the same (clonal) accessions of the largest olive collection held by the Research Centre for Olive, Fruit and Citrus Crops (CREA-OFA) in Italy; molecular, chemical and morphological descriptors were used to characterize the genetic diversity among and within olive accessions. SSR markers at 11 nuclear microsatellite loci were used for genotyping the complete collection of the olive germplasm [50]. The 15 cultivars used in the present study were chosen for their different geographical origin, from “Bianchera”, selected in the Friuli Venezia Giulia Region to “Nocellara del Belice”, selected in Sicily; some of the cultivars are known and cultivated all over the world, as “Coratina” and “Frantoio”, others only at a local scale, such as “Palmarola” (from Basilicata) and “Morchiaio” (from Toscana). The selected cultivars also differ in their fruit utilization purposes, as “S. Caterina” is suitable for green table olives, “Moraiolo” for oil production, whereas “Maiatica di Ferrandina” produces dual-purpose fruits, suitable for being processed for oil or sweetened fruits production. The list of cultivars used in the experiment is reported in Table 4, together with the different geographical origins, fruit utilization, and cultivation spread. 

### 4.2. Sample Collection

For each cultivar, 3 healthy clonal plants were selected; on the selected plants, 3 distinct samples of leaves were collected from the middle part of the bearing zone of the previous year’s shoots (vegetation 2020). The leaves were randomly taken from the canopy. The samples were collected on 16 September (T1), 20 October (T2), and 18 November (T3) of 2021. These sampling times were chosen as, in general, from August to November, there is a transition from a period of high temperatures and drought of the Mediterranean summer to a rainy period during which solar radiation and temperatures progressively decrease. All samples for each survey (75–90 g of healthy leaves for each cultivar) were collected the same day, on the same trees for T1, T2, and T3, stored in the fridge in plastic bags, and separated for different analyses the day after. For VOCs analysis, fresh and not-stored leaves were used.

### 4.3. Climatic Data

**Figure 7:**
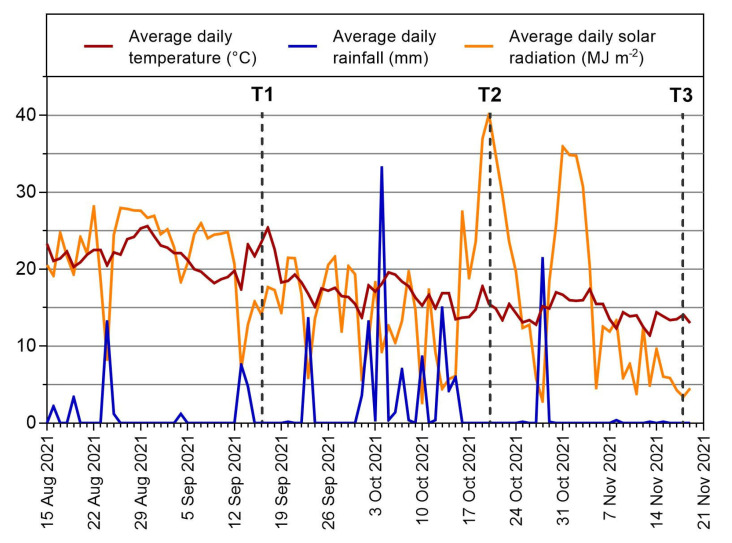
Climatic data (daily average temperature, rainfall, and solar radiation) recorded by the Montepaldi weather station from August to November 2021.

Climatic data (daily average temperature, rainfall, and solar radiation) were recorded by the Montepaldi weather station. The data collected from August to November 2021 are reported in Figure 7, relating to the whole sampling time. To make data comparable, surveys started on 15 August for all three environmental parameters considered. The first leaf sampling (T1) was carried out on 16 September, after a period characterized by reduced precipitations (average daily rainfall from 15 August to 15 September was about 1 mm), high solar radiation (22 MJm^−2^), and an average temperature of 21.7 °C. The second leaf sampling (T2) was performed on 20 October, after frequent rainfall events (average daily rainfall from 17 September to 19 October was 3.26 mm), solar radiation of 15.3 Wm^−2^, and average temperature of 17.2 °C. The third leaf sampling (T3) was carried out on 18 November, after a period of almost absent rainfall (average daily rainfall from 21 October to 17 November was 0.92 mm), solar radiation of 15.9 Wm^−2^, and average temperature of 14.5 °C. 

### 4.4. Leaf Gas Exchange Measurements

On the same days as the leaf samples’ collection, leaf gas exchange measurements were performed. The portable open gas exchange system Li-6400 XT (LiCor Inc., Lincoln, NE, USA) was used for the estimation of net photosynthetic rate (A_N_) and stomatal conductance (g_s_) on the previous year’s leaves. The measurements were taken between 10:00 a.m. and 2:00 p.m. on three different trees of each cultivar. Two undamaged healthy leaves from each plant were selected at different heights of the middle part of the previous year’s vegetation, in the south-east part of the canopy. The photosynthetic active radiation (PAR) resulted in 610, 880 and 450 µmol m^−2^s^−1^, for T1, T2, and T3, respectively. The photosynthetic parameters were determined with reference CO_2_ of 400 μmol mol^−1^, ambient relative humidity (40–50%), flow rate of 500 μmol s^−1^, chamber temperature of 25 °C, and photosynthetically active radiation of 700 μmol m^−2^ s^−1^, corresponding to a mean value of the light intensity during the sampling times.

### 4.5. Volatile Organic Compounds Analysis 

A commercial PTR-MS 8000 instrument, a useful tool for achieving the whole mass spectra from several vegetal matrices, was used as the detector for the organic compounds emitted from olive leaves belonging to different cultivar. Each analyzed sample consisted of 5 fresh leaves cut into 4 parts (crosswise) and subsequently introduced into a 500 mL glass container provided with a cover in which two teflon tubes was inserted, connected to a zero-air generator (Peak Scientific) and to the VOCs analyzer, respectively. Therefore, we wanted to assess the wound-related VOC emission and compare it to the different sampling times and cultivars. Moreover, since the chemical reactions are very sensitive to temperature variations, all the measurements were conducted inside a conditioned room with a temperature of 23 ± 1 °C. For each analyzed sample, the headspace analysis took place for 120 s with an acquisition rate of 1 spectrum per second using a mass spectrum range from 20 to 210 *m*/*z*. Before analyzing each sample, the jar was cleaned through the passage of “clean” air and the background noise was recorded by analyzing the empty jar (blank). For each cultivar, 3 leaf samples were analyzed for each sampling time and a total of 135 samples were evaluated (3 samples × 3 times × 15 cultivars). 

The ionization conditions of the “Drift Tube” were set as follows: voltage 600 V, temperature 60 °C, pressure 2.25 mbar, which resulted in E/N ratio of 140 Td (1 Td = 10^−17^ Vcm^−2^). The internal calibration of the instrument was based on three known compounds: *m*/*z* = 29.997 (NO^+^); *m*/*z* = 59.049 (C_3_H_7_O^+^), and *m*/*z* = 137.132 (C_10_H_17_^+^), and was performed offline. The raw mass spectral data, expressed as counts-per-second (cps), were acquired with the TofDaq Software (Tofwerk AG, Thun, Switzerland). The raw data of each sample (cps) were normalized according to what was stated by Jardine et al. (2010) [51] in normalized data (ncps = normalized count per second) on the basis of the primary ion signal. The volatile profile of “blank” was always subtracted from the final value of each sample.

### 4.6. Oleuropein Extraction and Quantification

Plant leaves were dried in an oven at 35 °C for 3 days and then pulverized. One gram of powder was solubilized in 10 mL of 1 mM HCl pH 3 and incubated at 60 °C for 4 h, shaking the sample every 30 min [8]. Finally, the samples were centrifuged at 12,000 rpm for 10 min and supernatants were filtered with a 0.22 μm filter. OL quantification was performed by RP-HPLC using a C18 column, 3 µm, 15 × 4.6 cm (Supelco, Bellefonte, PA, USA) and an OL standard was used for the calibration curves’ construction (Extrasynthese). Elution gradient was performed at a flow rate of 0.8 mL/min with the following solvent system: 10 mM trifluoroacetic acid (TFA) in acetonitrile (solvent A); 10 mM TFA in water (solvent B). The gradient used was 0% A for 2 min, from 0% to 12% A for 3 min, from 12% to 25% A for 25 min, from 25% to 100% A for 5 min, and detection was based on UV absorbance at 280 nm. Under these conditions, the OL peak appeared at a retention time (Rt) of 28.1 min. Samples of 10 µL were analyzed and quantification (mg g^−1^) was performed using the Chromeleon software.

### 4.7. Statistical Analyses

Two-way analyses of variance (ANOVA) were performed to determine if the two considered factors (15 cultivar and 3 sampling times, T1, T2, T3) had a statistically significant effect on the dependent variable. These analyses were performed using different data matrices, such as: (1) A_N_ (µmol CO_2_ m^−2^s^−1^); (2) g_s_ (mol m^−2^s^−1^); (3) OL content (mg g^−1^); and (4) total VOCs content (ncps). For each factor, the Fisher’s least significant difference (LSD) test was applied to highlight significant differences among cultivars and sampling times.

Computations were performed by Statgraphics Centurion XV v. 19.4.04. After a logarithmic transformation (log^+1^) and mean centering, principal component analysis (PCA, unsupervised method) was performed only on VOC data (average of triplicates), collected from 15 different cultivars and 3 different sampling times. Computations were performed by PLS-Toolbox v. 8.0.2 (Eigenvector Research Inc., West Eaglerock Drive, Wenatchee, WA, USA) for MATLAB R2015b (Mathworks Inc., Natick, MA, USA). 

To verify the influence of the climatic elements (temperature, rainfall, solar radiation) on the volatome (the set of masses that characterizes the emission of each individual genome), the data were submitted to canonical correspondence analysis (CCoA) [52], which is widely used in ecological data analysis [53], on a subset of VOCs with greater discriminant power among the surveys periods (T1, T2, and T3), previously selected on a statistical basis (significant univariate F-ratio at *p* = 0.01). During preprocessing, data were submitted to a logarithmic transformation (log^+1^). This method allows the comparison of two sets of variables, the extraction of ordination axes that are linear combinations of VOCs (criterion variables), and the explaining, at the same time, of as much of the variance as possible in the environmental data (explanatory variables). The samples were ordered with the components maximally interpreting the environmental data as well. Computations were performed by SYN-TAX 2000, Ordination package [54].

## 5. Conclusions

This study highlights, for the first time, the significant influence that climate elements, such as temperature, rainfall, and solar radiation, have on some responses of primary metabolism and, above all, on some phenomena related to secondary metabolism in a short time period. While the measured parameters related to the primary metabolism were much more genotype-dependent and also relatively stable in the succession of climate trends characterized by different rainfall intensity conditions, the phenomena related to the secondary metabolism were influenced by high quantitative variations. In the case of VOCs, the characteristics of the spectra profiles were also influenced, despite the analyses being carried out on homologous two-year-old leaves, and, therefore, being relatively stable. Among the considered climatic elements, rainfall seems to have the most influence on the parameters measured during the months of the test (September–November). After a period characterized by reduced rainfall (T1), a phase characterized by high rainfall (T2) caused a significant increase in the VOCs emission from wounded leaves, with different behaviors among the genotypes. Moreover, for their scientific and economic importance, the most interesting data are related to the OL concentration, which allowed the identification of both the cultivars with the highest concentration of this biophenol and the environmental conditions which could vary its level. In particular, the rainfall in the period before the sampling (T2) seemed to favor and increase the OL concentration in the two-year-old leaves, whereas the values measured after approximately the same period of drought (T1 and T3) were lower. The cultivar that presented the highest OL concentration was “Coratina”, followed by “S. Francesco”, “Itrana”, and “Maiatica di Ferrandina”. 

Further research and analyses will be needed to better verify both the distribution of the OL among leaves of different ages and cultivars, and the interactions between this compound and climate elements and fruit load. Such information, indeed, is fundamental for rationalizing the collection of leaves to be used as raw material for the extraction of antioxidant principles and for contributing to the enhancement of the cultivation of the olive tree thanks to the multiplicity of use of its products.

## Figures and Tables

**Table 1:** Summary of the two-way ANOVA results for (a) net photosynthetic rate (A_N_, µmol CO_2_ m^−2^s^−1^) and (b) stomatal conductance (g_s_, mol m^−2^s^−1^) obtained from 15 olive cultivars at the 3 sampling times (T1, T2, T3). Different lowercase letters indicate the differences among the average A_N_ or g_s_ values of all the cultivars at the three sampling times (T1, T2, T3) by the LSD test at the 95.0% confidence level (*p* = 0.05). All F-ratios are based on the residual mean square error.

Source	Sum of Squares	Effect (%)	Df	Mean Square	F-Ratio	*p*-Value
Net photosynthetic rate (A_N_)						
Main effect						
A: Time	45.29	10.55	2	22.65	91.82	<0.0001
B: Cultivar	263.98	61.48	14	18.85	76.44	<0.0001
Interactions						
AB	97.89	22.80	28	3.49	14.17	<0.0001
Residual	22.20	5.17	90	0.24		
Total (Corrected)	429.37	100	134			
Average A_N_ (µmol CO_2_ m^−2^s^−1^)	Time 1		Time 2		Time 3	
	12.60 b		11.30 a		11.40 a	
Stomatal conductance (g_s_)						
Main effect						
A: Time	0.016	12.16	2	0.0084	51.39	<0.0001
B: Cultivar	0.056	40.29	14	0.0040	24.32	<0.0001
Interactions						
AB	0.051	36.90	28	0.0018	11.14	<0.0001
Residual	0.014	10.65	90	0.00016		
Total (Corrected)	0.13	12.16	134			
Average g_s_ (mol m^−2^s^−1^)	Time 1		Time 2		Time 3	
	0.16 a		0.18 b		0.18 b	

**Table 2:** Summary of the two-way ANOVA results for VOCs emission amount (ncps) obtained from 15 olive cultivars at the 3 sampling times (T1, T2, T3). Different lowercase letters indicate the difference among the average VOCs emission values of all the cultivars at the three sampling times (T1, T2, T3) by the LSD test at the 95.0% confidence level (*p* = 0.05). All F-ratios are based on the residual mean square error.

Source	Sum of Squares	Effect (%)	Df	Mean Square	F-Ratio	*p*-Value
Main effect						
A: Time	7.46 × 10^10^	33.38	2	3.73 × 10^10^	212.45	<0.0001
B: Cultivar	3.34 × 10^10^	14.95	14	2.38 × 10^9^	13.59	<0.0001
Interactions						
AB	9.97 × 10^10^	44.59	28	3.56 × 10^9^	20.27	<0.0001
Residual	1.58 × 10^10^	7.07	90	1.75 × 10^8^		
Total (Corrected)	2.23 × 10^11^	100	134			
Average VOCs emission (npcs)	Time 1		Time 2		Time 3	
	12,996 a		68,299 c		54,615 b	

**Table 3:** Summary of the two-way ANOVA results for oleuropein concentration (mg g^−1^) obtained from 15 olive cultivars at the 3 sampling times (T1, T2, T3). Different lowercase letters indicate the difference among the average oleuropein concentration values of all the cultivars at the three sampling times (T1, T2, T3) by the LSD test at the 95.0% confidence level (*p* = 0.05). All F-ratios are based on the residual mean square error.

Source	Sum of Squares	Effect (%)	Df	Mean Square	F-Ratio	*p*-Value
Main effect						
A: Time	23,523.20	38.62	2	11,761.60	508.57	<0.0001
B: Cultivar	16,938.70	27.81	14	1209.91	52.32	<0.0001
Interactions						
AB	18,370.70	30.16	28	656.09	28.37	<0.0001
Residual	2081.42	3.42	90	23.12		
Total (Corrected)	60,914.00	100	134			
Average OL (mg g^−1^)	Time 1		Time 2		Time 3	
	11.90 a		40.00 b		12.10 a	

**Table 4:** List of cultivars used in the experiment with the corresponding geographical origin, cultivation spread, and fruit utilization.

Sample No	Name	Geographical Origin	Cultivation Spread	Product Use
1	Ascolana tenera	Marche	National	Table
2	Bianchera	Friuli Venezia Giulia	Local	Oil
3	Carolea	Calabria	National	Oil/Table
4	Coratina	Apulia	International	Oil
5	Frantoio	Tuscany	International	Oil
6	Itrana	Lazio	National	Oil/Table
7	Leccino	Tuscany	International	Oil
8	Maiatica di Ferrandina	Basilicata	Local	Oil/Table
9	Moraiolo	Tuscany	National	Oil
10	Morchiaio	Tuscany	Local	Oil
11	Nocellara del Belice	Sicily	National	Oil/Table
12	Palmarola	Apulia	Local	Oil/Table
13	S. Agostino	Apulia	International	Table
14	S. Caterina	Tuscany	Local	Table
15	S. Francesco	Tuscany	Local	Table

## Data Availability

The data presented in this study are available on request from the corresponding author.
